# Factors Affecting the Efficiency of Near-Infrared Indocyanine Green (NIR/ICG) in Lymphatic Mapping for Colorectal Cancer: A Systematic Review

**DOI:** 10.7759/cureus.55290

**Published:** 2024-02-29

**Authors:** Irina Shevchenko, Dragos Serban, Ana Maria Dascalu, Laura Tribus, Catalin Alius, Bogdan Mihai Cristea, Andra Iulia Suceveanu, Daniel Voiculescu, Dan Dumitrescu, Florin Bobirca, Adrian Paul Suceveanu, Dragos Eugen Georgescu, Crenguta Sorina Serboiu

**Affiliations:** 1 Surgery, Faculty of Medicine, Carol Davila University of Medicine and Pharmacy, Bucharest, ROU; 2 General Surgery, Emergency University Hospital Bucharest, Bucharest, ROU; 3 Ophthalmology, Faculty of Medicine, Carol Davila University of Medicine and Pharmacy, Bucharest, ROU; 4 Gastroenterology, Faculty of Oral Medicine, Carol Davila University of Medicine and Pharmacy, Bucharest, ROU; 5 Anatomy, Faculty of Medicine, Carol Davila University of Medicine and Pharmacy, Bucharest, ROU; 6 Gastroenterology, Faculty of Medicine, Ovidius University of Constanta, Constanta, ROU; 7 Gastroenterology, Clinical Emergency Hospital St Apostle Andrew, Constanta, ROU; 8 General Surgery, Clinic Hospital "Dr. Ioan Cantacuzino" Bucharest, Bucharest, ROU; 9 Medicine, Faculty of Medicine, Ovidius University of Constanta, Constanta, ROU; 10 Radiology, Faculty of Medicine, Carol Davila University of Medicine and Pharmacy, Bucharest, ROU

**Keywords:** intraoperative dissection, lymphatic mapping, predictive factors, colorectal cancer, indocyanine green, near infrared fluorescence

## Abstract

As laparoscopy gained global popularity in oncologic surgery, the challenge of detecting lymph nodes spurred researchers to explore innovative techniques and approach the situation from a fresh perspective. While many proposed methods have faded into obscurity, the utilization of indocyanine green (ICG) in the surgical treatment of oncologic patients has continued to advance. The immense potential of this dye is widely acknowledged, yet its full extent and limitations in lymphatic mapping for colorectal cancer remain to be precisely determined. This article aims to assess the magnitude of its potential and explore the constraints based on insights from clinical studies published by pioneering researchers. A systematic review of the existing literature, comprising articles in English, was conducted using the Scopus, PubMed, and Springer Link databases. The search employed keywords such as "colorectal cancer" AND/OR "indocyanine green," "fluorescence" AND/OR "lymphatic mapping" AND/OR "lymph nodes." Initially identifying 129 articles, the application of selection criteria narrowed down the pool to 10 articles, which served as the primary sources of data for our review.

Despite the absence of a standardized protocol for the application of ICG in colorectal cancer, particularly in the context of lymphatic mapping, the detection rates have exhibited considerable variation across studies. Nevertheless, all authors unanimously regarded this technique as beneficial and promising. Additionally, it is advocated as an adjunctive tool to enhance the accuracy of cancer staging. Near-infrared (NIR)-enhanced surgery holds the promise of transforming the landscape of oncologic surgery, emerging as a valuable tool for surgeons. However, the absence of a standardized technique and the subjective nature of result assessment impose limitations on the potential of this method.

Consequently, it can be inferred that the establishment of a universally accepted protocol, encompassing parameters such as dose, concentration, technique, and site of administration of ICG, along with the optimal time needed for fluorescence visualization, would enhance the outcomes. Emphasizing the accurate selection of patients is crucial to prevent the occurrence of false-negative results.

## Introduction and background

Colorectal cancer (CRC) stands as a notable worldwide health challenge, contributing significantly to the annual toll of cancer-related fatalities. The World Health Organization (WHO) has identified CRC as the third most prevalent oncological ailment, responsible for 930,000 deaths each year [1,2]. The prevalence of CRC is on the rise, attributed to various factors like an aging population, obesity, dietary patterns, smoking, and insufficient physical activity [3-6]. Consequently, there is an escalating need for heightened focus and exploration of novel approaches to its management, encompassing surgical interventions.

A pivotal moment in the realm of oncological surgery can be identified with the observations made by the German pathologist Rudolf L.K. Virchow, who noted that lymph nodes (LNs) act as filters for particulate matter in lymph fluid and that cancer spreads through lymph ducts to reach the LNs and emergence of two key theories based on these findings, setting the course for subsequent research directions [7,8].

During the 19th century, William Halsted introduced a theory positing a sequential development of cancer progression, suggesting the predictability of metastatic spread. However, this hypothesis was later contradicted by Fisher, who asserted that metastases spread in a random and unpredictable manner [9,10].

These theories laid the foundation for the development of two primary approaches in the management of patients with CRC: limited colonic resection and complete mesocolic excision with central vascular ligation but both methods have proponents and critics [11-13].

It is evident that the excision of LNs infiltrated by tumor cells is a crucial aspect of the surgical treatment of CRC, aimed at preventing cancer spread and recurrence [14,15]. The American Joint Committee on Cancer (AJCC) Cancer Staging Manual highlights that the presence of LN metastases nearly doubles the risk of local recurrence [16,17]. Furthermore, the National Comprehensive Cancer Network (NCCN), the College of American Pathologists, and the AJCC have collectively recommended that resected specimens should ideally contain a minimum of 12 LNs for adequate N staging [18,19].

This resulted in numerous minimally invasive surgical approaches that have been put forth for CRC over the past decades, aimed at reducing postoperative complications and enhancing patient survival and has culminated in the emergence of specific areas of interest, notably fluorescence imaging [20,21]. Fluorescence imaging offers real-time guidance to surgeons across various procedures, holding the potential to revolutionize human surgery [22,23].

Despite the various imagistic tools proposed for intraoperative navigation and LN detection, indocyanine green (ICG) continues to be regarded as a promising and safe solution for the surgical management of cancer due to its unique properties.

At present, ICG finds application in oncologic surgery for tasks such as intraoperative tumor localization, assessment of perianastomotic vascularization, and lymphatic mapping [24,25].

ICG is an approved tricarbocyanine iodide dye for clinical use. It exhibits high water solubility, amphiphilic characteristics, and a propensity to bind to plasma proteins, particularly lipoproteins. When exposed to near-infrared (NIR) light, it fluoresces with excitation and emission wavelengths of 780 nm and 820 nm, respectively [26,27]. Notably, it possesses a favorable signal-to-noise ratio and excellent tissue penetration. Metabolized in the liver and excreted with bile without intestinal absorption, it demonstrates low toxicity [28,29]. These distinctive properties enable rapid circulation in the bloodstream, leading to accumulation in lymphatic pathways and regional LNs [30].

Through an analysis of existing literature, this review aims to furnish information on the safety of lymphatic mapping using ICG, along with the sensitivity, specificity, and accuracy of ICG fluorescence in assessing LN metastasis. Additionally, it will explore the technical features and perspectives of this method.

## Review

Materials and methods

A comprehensive search of the PubMed, SpringerLink, and Scope databases was conducted until October 2023 to identify pertinent articles related to lymphatic mapping in CRC. The search employed index terms such as "colorectal cancer" AND/OR "indocyanine green," "fluorescence" AND/OR "lymphatic mapping" AND/OR "lymph nodes." The literature search adhered to the Preferred Reporting Items for Systematic Reviews and Meta-Analysis (PRISMA) guidelines [31].

The PICO (Population, Intervention, Comparison, Outcome) format was employed to identify eligible studies to be included in the review: P (Population): Individuals diagnosed with CRC regardless of the stage; I (Intervention): Surgical treatment of CRC with the use of ICG for lymph node identification; C (Comparison): Traditional approach for lymph node identification without ICG and using NIR fluorescence; O (Outcome): Quantification of both harvested and positive lymph nodes.

**Figure 1:**
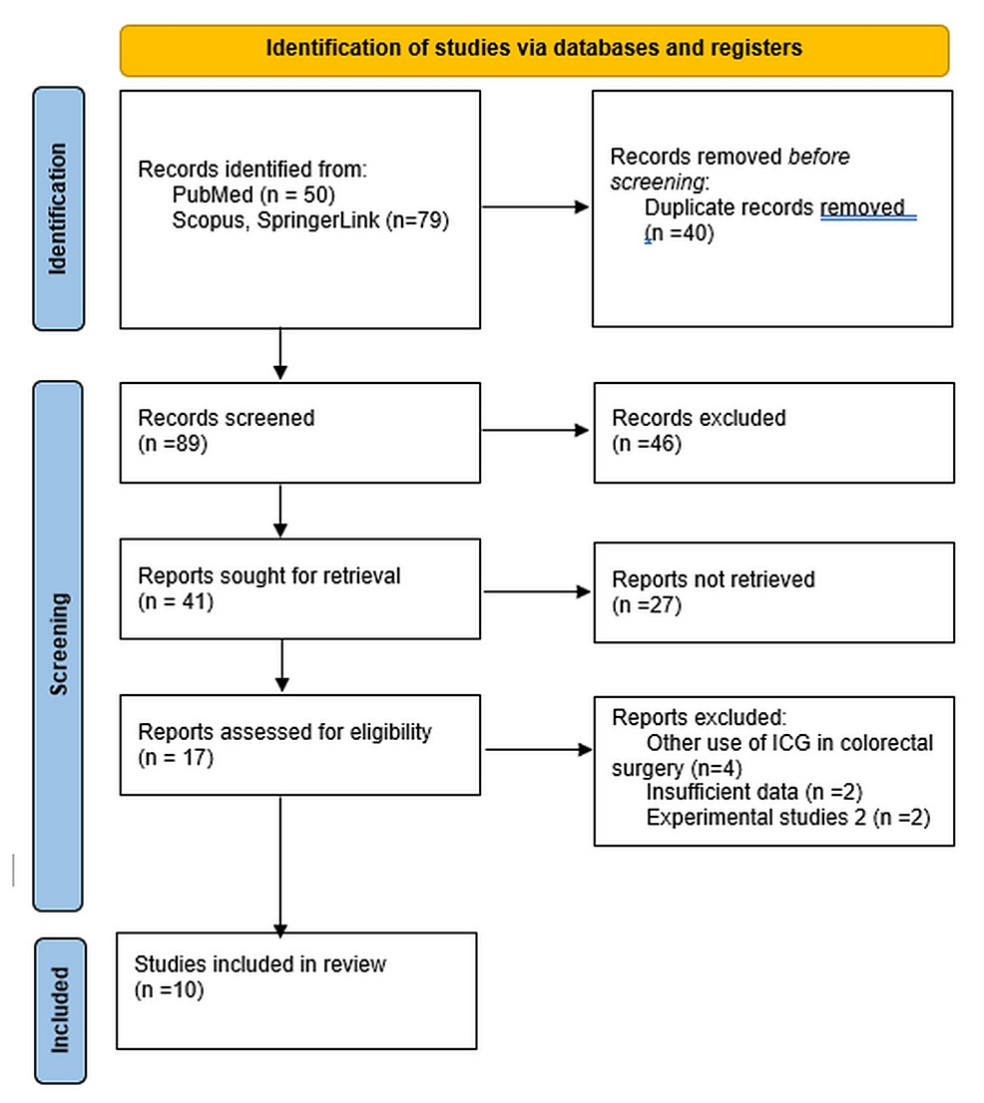
Preferred Reporting Items for Systematic Reviews and Meta-Analysis (PRISMA) for the studies included in the review ICG: indocyanine green

Inclusion criteria comprised full-text studies in English with a minimum sample size of 10 enrolled patients, detailed descriptions of the lymphatic mapping technique, and the inclusion of human patients with CRC (Figure 1).

Our review excluded articles describing clinical cases, with fewer than 10 patients enrolled, those with incomplete descriptions of the technique, publications in languages other than English, and studies detailing the use of ICG in types of cancer other than colorectal and/or rectal cancer.

The extracted information encompassed clinicopathological characteristics, underlying pathology, first author, year of publication, ICG concentration/dosage, time and site of ICG injection, average number of extracted LNs, average number of positive LN, detection rate, and identification of LNs outside the standard lymphadenectomy boundaries.

To assess data quality, the methodological index for non-randomized studies (MINORS) score was employed and is presented in Table 1. Among the nine prospective studies incorporated in this review, the median MINORS score [32] was 15 out of 16 for noncomparative studies and 20 out of 20 for one randomized study that was evaluated.

**Table 1:** Methodological index for non-randomized studies (MINORS) for the reviewed studies

MINORS score	Chand et al. 2018 [33]	Sato et al. 2021 [34]	Picchetto et al. 2023 [35]	Ushijima et al. 2020 [36]	Wan et al. 2022 [37]	Nagata et al. 2006 [38]	Ribero et al. 2022 [39]	Watanabe et al. 2016 [40]	Cao et al. 2021 [41]	Currie et al. 2017 [42]
A clearly stated aim	1	1	2	2	2	2	2	2	2	2
Inclusion of consecutive patients	2	2	2	2	2	2	1	2	2	2
Prospective collection of data	2	2	2	2	2	2	2	2	2	2
End points appropriate to the aim of the study	2	1	2	2	2	2	2	2	2	2
Unbiased assessment of the study end point	2	2	2	2	2	2	2	2	2	2
Follow-up period appropriate to the aim of the study	2	2	2	2	2	2	2	2	2	2
Loss to follow-up <5%	2	2	2	2	2	2	2	2	2	2
Prospective calculation of the study size	2	2	2	2	2	2	1	2	2	2
An adequate control group	-	-	-	-	2	-	-	-	-	-
Contemporary groups	-	-	-	-	2	-	-	-	-	-
Baseline equivalence of groups	-	-	-	-	2	-	-	-	-	-
Adequate statistical analyses	-	-	-	-	2	-	-	-	-	-
TOTAL	15/16	14/16	16/16	16/16	24/24	16/16	14/16	16/16	16/16	16/16

Results

We conducted a systematic review that encompassed 10 studies with a total enrollment of 591 patients, investigating lymphatic mapping in CRC [33-42].

The sample sizes across the studies ranged from 10 to 155 patients, with a cumulative enrollment of 591 [33-42]. There was no significant difference observed in average body mass index (BMI), which ranged from 22.5 to 26.2. BMI was not reported in two out of the 10 studies [33,35]. The majority of patients in each study were men, with the exception of the study by Chand et al. [33], which reported a male-to-female ratio of 40% vs 60%.

The average age exhibited notable variation among studies, ranging from 58 to 71.5 years [33,34]. One author focused on the lymphatic flow of left colon cancer, while six studies also included cases of rectal cancer [35-41].

The selected articles are detailed in Table 2, and clinicopathologic data extracted from these publications is presented in Table 3.

**Table 2:** Clinicopathologic characteristics of the patients included in the reviewed studies BMI: body mass index; TNM stage: TNM classification system of malignant tumors; Mt: metastasis; M/F: male/female

Author	Age	BMI	TNM Stage	Distant Mt	Tumor site	M/F
Chand et al. 2018 [33]	69.5	-	T1-T3/N1/N2	0	right/left/sigmoid	40%; 60%
Sato et al. 2021 [34]	66	22.8	T1-T4/N0/N+	6.5%	right/left colon, rectum	53.5; 46.5%
Picchetto et al. 2023 [35]	-	-	-	-	colon/rectum	-
Ushijima et al. 2020 [36]	71.5	22.9	T1-T4/N0/N+	-	right/left/sigmoid colon	57.9%; 42.1%
Wan et al. 2022 [37]	58	23.4	T1-T4a/N0/N+	0	sigmoid/rectum	63.6%; 36.4%
Nagata et al. 2006 [38]	63.9	22.5	T1-T3/N0/N+	0	right/left/sigmoid colon, rectum	58.3%; 41.7%
Ribero et al. 2022 [39]	63	25.3	Tis-T3/N0/N+	0	right/left colon, rectum	54.3%; 45.7%
Watanabe et al. 2016 [40]	67.5	23.6	Tx/N0	0	left colon	71%; 29%
Cao et al. 2021 [41]	65	23.4	T2-T4/No/N+	-	colon, rectum	91%; 9%
Currie et al. 2017 [42]	69	26.2	T1- T2/N0	0	right/left colon	-

**Table 3:** Clinical studies regarding lymphatic mapping using indocyanine green (ICG) No.: number; NA: not acknowledged; iv: intravenous

Author/year	No. of patients	ICG concentration	ICG volume	Time of ICG injection	Site of ICG injection	Type of surgery	Imaging system	In vivo/ex vivo	No. of harvested lymph nodes (mean)	No. of positive lymph nodes	Lymph node detection rate	Change in intraoperative management	ICG side effects
Chand et al. 2018 [33]	10	0.5-1.6 mg/ml	4 ml	Intraoperative	Subserosal	Laparoscopic	laparoscopic fluorescence camera systems (PinpointTM, Novadaq AIM 1588TM, Stryker) AIM 1588TM, Stryker	Ex vivo	22	2	100%	20%	None
Sato et al. 2021 [34]	155	2.5 mg/ml	0.1 ml	Preoperative	Submucosal	Laparoscopic	AIM camera system	In vivo / ex vivo	20.3	7.9	3.2%	-	None
Picchetto et al. 2023 [35]	146	0.1-5 mg/ml	0.4 ml-6 ml	perioperative/intraoperative	Subserosal submucosal	Laparoscopic	D-Light-P Karl Storz, SPY Stryker, PINPOINT Novadaq, VISERA ELITE Olympus, Artemis Spectrum	In vivo/ex vivo	-	-	72.5%	50%	None
Ushijima et al. 2020 [36]	57	2.5 mg/ml	0.2-0.3 ml	24-48 h	Submucosal	Laparoscopic	PINPOINT NIR laparoscopic camera system		25	222	75.4%	-	None
Wan et al. 2022 [37]	33	2.5 mg/ml	4-12 ml	24 h	Submucosal	Laparoscopic	fluorescence imaging equipment (Storz)	In vivo	28	0.05	100%	-	None
Nagata et al. 2006 [38]	48	5 mg/ml	5 ml	Intraoperative	Subserosal	Laparoscopic	-	NA	21	-	87.5%	-	None
Ribero et al. 2022 [39]	70	0.5 mg/ml	1.5 ml/injection; 4 injections	24-72 h	Submucosal	Robotic	da Vinci Xi platform	In vivo	18	3	92.8%	50%	None
Watanabe et al. 2016 [40]	31	2.5 mg/ml	2 ml	Intraoperative	Subserosal submucosal	Laparoscopic	The laparoscopic NIR camera system was provided by Karl Storz	In vivo/ex vivo	17.5	10.4	100%	-	None
Cao et al. 2021 [41]	11	25 mg/30 ml	30 ml	24 h	iv	Laparoscopic	FLI-10B fluorescence navigation system	Ex vivo	40	38	95%	-	None
Currie et al. 2017 [42]	30	5 mg/ml	1 ml x 4	Intraoperative	Submucosal	Laparoscopic	prototype Laparoscopic Near-Infrared Fluorescence Imaging System Olympus	In vivo	34	1	97%	-	None

Technical Aspects of ICG Injection: Dose, Concentration, Technique

Prior to delving into specific aspects of various studies, we conduct critical assessments encompassing all studies. The majority of authors reported laparoscopic approach as a surgical treatment method and standardized approach [33-38,40-42], except Ribero et al. [39] who performed robotic colorectal resection using da Vinci® Xi platform (Intuitive Surgical, Inc., Sunnyvale, CA, USA). In one study associated laparoscopic hepatectomy was effectuated for metastasis.

The concentration of administered ICG in the included studies exhibited significant variation, ranging from 0.1 to 5 mg/ml [40,42]. Moreover, the volume of administered ICG varied widely, spanning from 0.1 to 30 ml [33,41]. The injection sites were both submucosal and subserosal, administered intra- and perioperatively.

In 2006, Nagata et al. [38] reported a series involving 48 patients with CRC, utilizing ICG as a tracer for lymphatic mapping and sentinel lymph node (SLN) detection before mesenteric dissection. A total volume of 5 ml of ICG with a concentration of 5 mg/ml was subserosally injected proximal and distal to the tumor during surgery following saline injection (as the authors noted that saline injection increased mapping accuracy) [37]. A similar technique was described by Chand and colleagues in 2018 [33], where ICG was also introduced subserosally, but with a smaller volume and concentration (0.5-1.6 mg/ml, 4 ml) after the specimen was extracorporealized [33]. Five other authors preferred submucosal injection of the fluorescent substance using preoperative or on-table colonoscopy [34,36,37,39,42]. Despite the similarity in the procedure for introducing indocyanine in these studies, the timing of use varied significantly. For instance, Currie and colleagues [42] described the intraoperative use of ICG, incorporating colonoscopy as a stage of the operation [42]. On the other hand, other authors preferred a time window of 24-72 hours preoperatively [33,35,36,38], most often in a concentration of 2.5 mg/ml.

Watanabe et al. [40] and Picchetto et al. [35] specified both submucosal and subserosal injection sites intra- and perioperatively. In 2021, Cao and colleagues [41] demonstrated intravenous administration of 25 mg/30 ml of ICG before surgery and mentioned four time points (30 minutes, one hour, two hours, four hours) for tumor and LN detection. Notably, no complications related to the technique were reported by the authors.

Detection of Metastatic LNs in CRC Using NIR-ICG

**Figure 2:**
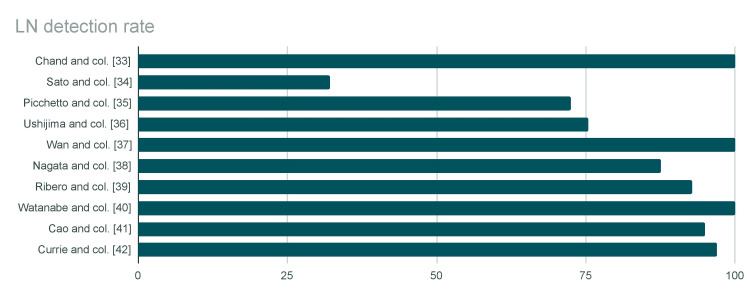
Lymph node (LN) detection rate in the reviewed studies

The detection rates exhibited considerable variation across studies, ranging from 32.2% to 100% (Figure 2).

The detection rate of LNs was not influenced by patients' BMI or tumor site or differentiation among the reviewed studies.

When compared to standard laparoscopy, all authors found that identification of the SLN is highly superior when NIR ICG technique was employed, being a valuable tool to describe the lymph flow in 87.5-100% of cases [34,38,40]. This finding could be of significant clinical benefit especially in challenging locations, such as splenic flexure cancer [40], with highly individual anatomical variations.

Detecting metastatic LN by using ICG imaging was found to be variable according to the tumoral stage. Using the NIR-ICG detection technique for metastatic LNs was reported to have excellent results in the case of T1-T2 stage, and no false-negative results were reported [38].

However, false-negative results were identified in advanced tumors, with sizes exceeding 35 mm and pT3-T4 stages, due to LN obstruction by tumor cells, leading to changes in lymph drainage through the development of alternative pathways [34,36,38,42].

Many authors underscored the correlation between tumor stage and the efficacy of fluorescence detection. Sato et al. [34], Ushijima et al. [36], Nagata et al. [38], and Currie et al. [42] collectively reached a consistent conclusion, asserting that the utility of this LN identification method is diminished in advanced stages of CRC. These observations stand in contrast to the findings of Cao et al. [41] and Chand et al. [33], who reported strong fluorescence in LNs harboring metastases. Chand et al. also identified various factors affecting visualization during the procedure.

Regarding the role of ICG in extending the LN dissection over the standard resection boundaries, the results are conflicting. While Currie et al. [42] found no fluorescent LNs outside the standard areas, other studies revealed that using NIR ICG in detecting LNs modified the surgical approach in a significant number of cases [33,35,37,39]. Pichetto et al. [35] and Ribero et al. [39] found that LN dissection was extended in over 50% of cases upon the standard limits according to the tumor localization when staining with ICG was used. Harvesting more potentially metastatic LNs could be a valuable factor in improving survival in these patients. Moreover, in a study on 10 patients with rectosigmoidian cancer, Chand et al. [33] found two cases (20%) with fluorescent LN surpassing the standard dissection area, prompting the extension of resection margins. The resected LNs were positive on histopathology [33]. ICG imaging proved beneficial for the resection of LNs along the inferior mesenteric artery (D3), with paraaortic LNs visualized during the procedure and included in the dissection. These LNs could not be identified by normal laparoscopic white-light imaging [37].

A novel approach was used by Sato et al. [34] who incorporated microscopic ICG fluorescence imaging for enhanced LN detection and arrived at a similar conclusion that ICG fluorescence does not correlate with LN metastasis. Interestingly, heavily affected LNs did not exhibit fluorescence, with metastases detected in 2.8% of ICG-positive LNs and 5.8% of negative LNs. The authors noted that microscopically, both affected and normal LNs displayed fluorescence under infrared light. However, fluorescence was only observed at the site with normal morphology, while the region affected by cancer metastases did not exhibit fluorescence. The highest fluorescence was observed at the level of paracortex and medulla. Microscopic fluorescence could be detected if at least 10% of the LN morphology was maintained.

Discussion

A systematic review encompassing 10 studies involving 591 patients on ICG-guided colorectal surgery suggests that ICG could be beneficial. However, certain weaknesses persist in this field.

Technique of Lymphatic Mapping in CRC Using ICG

In CRC, ICG finds applications in ureteral identification, the evaluation of peritoneal or hepatic metastasis, assessment of perianastomotic perfusion, tumor visualization, assessment of resection margins and lymphatic mapping. Consequently, various methods of ICG administration are employed [43-45].

While numerous authors have proposed diverse protocols for ICG dosage, timing, and administration methods, there is currently no universally accepted scheme in this field [46]. Even within the studies included in our review, significant differences in the technical aspects of ICG application were noted.

Although it is established that for lymphatic system evaluation, ICG should be injected into the colonic wall to drain through lymphatic vessels and accumulate in LNs, the optimal injection site remains a subject of debate. Some authors advocate for preoperative submucosal ICG injection, citing lower false negative results and the potential for dye spillage from lacerated lymphatics during intraoperative ICG injection [27]. However, the submucosal approach has been shown to be more expensive and associated with a lower general satisfaction score among patients due to the preoperative physical and mental load related to endoscopy [47].

The optimal timing for LN visualization is highly dependent on the injection technique. Interestingly, the best-reported time frame for lymphatic mapping after subserosal intraoperative ICG injection is 30-60 minutes [48], even though fluorescence persisted for 10 days in the case of preoperative submucosal ICG administration for tumor localization and for 72 hours for lymphatic mapping [38,49].

Regarding dosage, a dosage range of 0.5-1 mg of ICG was associated with the highest detection rate irrespective of the purpose [23]. However, our review did not reveal any consistent relationship between the dosage of ICG used in studies and the LNs' detection rate.

Factors Influencing the Effectiveness of the Lymphatic Mapping Using NIR/ICG

Lymphatic mapping and SLN procedures in CRC are gaining popularity and undergoing thorough investigation [50-55]. However, existing studies have reported controversial results [48,49]. Due to the ambiguous data presented in current publications and the lower efficiency of ICG in CRC compared to other types of neoplasia, the evaluation of lymph flow and detection of LNs using ICG is recommended for better staging of patients but cannot be considered a reliable tool for therapeutic decision-making [50-55].

In our systematic review, which included 10 articles containing data regarding lymphatic mapping in CRC, all studies underwent evaluation using the MINORS score for quality assessment. The sensitivity of ICG in identifying LNs varied from 32.2% to 100% (Table 3). No consistent relationship was found between ICG dosage, timing/site of injection, or tumor site. However, some authors reported a significantly higher rate of LN identification in cases of preoperative submucosal ICG injection [51,56,57].

An important observation was the variation in terminology used by authors, introducing subjectivity to results evaluation. Some studies described lymph flow evaluation with or without LN detection, while others mentioned SLN mapping [35,37,39,40,42]. The generally accepted definition of a SLN implies the first node receiving lymph directly from the tumor [58]. On the other hand, there is no clear definition of lymphatic mapping, but in the accepted sense, it refers to the imaging of the lymphatic network, including LNs and connecting lymphatic vessels where lymphatic flow is a mandatory part of the map [59].

The main finding of our study was a strong correlation between tumor stage/size and the efficiency of lymphatic mapping, consistent with other publications [33,37,42,57,60,61]. It was demonstrated that ICG has no specific tropism for metastasis, except in hepatocellular carcinoma, and lymph flow visualization is possible only due to ICG binding to albumin, which is high in lymph [52,62]. Therefore, LNs obstructed by metastatic tissue may not exhibit fluorescence due to the absence of lymph flow through these nodes. Even the peritumoral lymphatic network may be blocked by metastatic emboli or tumor-induced lymphangiogenesis might affect the morphology of the lymphatic system [63-65]. In such cases, alternative lymph flow develops, making fluorescence extremely helpful [33,66]. The mapping using ICG might be less efficient in patients after neoadjuvant chemotherapy and radiotherapy due to fibrosis and atrophy of LNs [38,67-69].

The variability in results can be clarified by considering technical aspects, tumor localization, the inclusion of patients in advanced stages, and the utilization of the in vitro method by certain researchers.

The current method's relatively low sensitivity constrains its extensive adoption in oncological surgery.

Future Directions in Using ICG in CRC

Early detection remains a key element in CRC, which ensures the best response to therapy and survival. According to the literature, further investigation has been conducted into the potential clinical practice implementation of deep learning algorithms for the classification and diagnosis of CRC histopathology images. The advancement made possible by deep learning algorithms has the potential to improve CRC detection's accuracy and efficacy [70,71].

ICG has proven effective in surgery as a non-targeting agent for identifying tumors and metastasis. However, it possesses certain undesirable properties such as low photostability, poor quantum yield, rapid elimination, and low specificity [71,72]. To enhance the productivity of ICG, various solutions have been proposed. One approach involves the use of ICG nanoparticles, where the ICG molecule is bound to different types of nanoparticle structures. This method aims to improve the physicochemical properties of ICG, enhance its stability, extend its half-life, and enable specific tumor binding [73].

Another strategy mentioned in the literature is the conjugation of ICG to antibodies. When ICG is linked to antibodies, it loses its fluorescent properties and regains them upon separation. Consequently, ICG conjugated to antibodies specific for tumor cells activates fluorescence after internalization in tumor cells [74,75]. Ogawa et al. reported a high quenching capacity of antibody-ICG complexes using antibodies such as daclizumab, panitumumab, and trastuzumab [76]. In a 2016 study, Harlaar et al. [77] conducted a non-randomized trial involving patients with peritoneal carcinomatosis of colorectal origin undergoing cytoreductive surgery. A fluorescent tracer, bevacizumab-IRDye800CW, was intravenously injected a few days before the surgical intervention. This method facilitated the detection of tumor tissue initially overlooked by surgeons. Lesions without fluorescence were found to be non-malignant, while 53% of fluorescent areas were identified as zones of adenocarcinoma [77]. The insights gained from this review hold significant implications for CRC management, potentially enhancing the precision of LN staging and guiding treatment decisions.

## Conclusions

Lymphatic mapping using ICG has the potential to redefine conventional boundaries and elevate the paradigm of oncological surgery to unprecedented heights. It envisions a scenario where the prognosis for cancer patients is no longer daunting or disheartening. Regrettably, the absence of a standardized protocol, the subjective nature of result assessment, and the lack of a robust evidence base with specific outcomes preclude an unequivocal conclusion regarding the efficacy of using ICG for LN detection as a dependable tool for surgeons.

Conversely, through careful patient selection and employing optimal methods for the application of this substance, remarkable and unforeseen results can be attained.
